# Conservative therapy of severe acute pancreatitis is a safe option – results of a 15-year long-term follow-up cohort study

**DOI:** 10.1097/MS9.0000000000000697

**Published:** 2023-04-18

**Authors:** Guido Alsfasser, Ernst Klar, Judith Feitl, Clemens Schafmayer

**Affiliations:** Department of General, Visceral, Thoracic, Vascular and Transplantation Surgery, University of Rostock, Rostock, Germany

**Keywords:** acute pancreatitis, conservative treatment, long-term follow-up

## Abstract

Treatment of severe acute pancreatitis can be challenging with high mortality. In 2012 we reported a significantly reduced in-hospital mortality if these patients are treated conservatively for at least the first 3 weeks in the course of the disease as compared to early necrosectomy. We performed a long-term follow-up and compared the outcome of these two study groups (group 1 – early necrosectomy, *n*=20 versus group 2 – primary conservative treatment, *n*=24).

**Materials and methods:**

Follow-up of the study patients by personal contact, phone survey, or data from primary care physician. Median follow-up was 15 years (range 10–22 years). This trial is registered at: Research Registry UIN researchregistry8697.

**Results:**

Eleven survivors of group 1 and 22 survivors of group 2 were discharged after initial treatment. Ten of 11 surviving patients of group 1 (90.9%) and 20 of 22 surviving patients of group 2 (90.9%) were included in this study. Between groups, there were no statistical differences in the rate of resubmission (*P*=0.23), development of diabetes (*P*=0.78), or development of exocrine insufficiency (*P*=1.0). However, long-term survival in group 2 was significantly better than that of group 1 (*P*=0.049).

**Conclusion:**

Primary conservative treatment of severe acute pancreatitis without early necrosectomy does not lead to early complications and even shows an advantage in long-term survival. Therefore conservative treatment of severe acute pancreatitis is safe and there is no absolute need for necrosectomy in severe acute pancreatitis.

## Introduction

Highlights

- Long-term follow-up after severe acute pancreatitis with a median follow-up of 15 years.
- Primary conservative therapy without necrosectomy does not result in more readmissions.
- Necrosectomy in severe acute pancreatitis can be avoided.

Severe acute pancreatitis is defined as the presence of organ failure for more than 48 h^1^. Many studies showed increased mortality in the treatment of severe acute pancreatitis when operations were performed early in the course of the disease. Mortality is indeed decreased when operations or interventions are performed late in the course of the disease^2^,^3^. Therefore a more conservative approach is recommended^4^–^7^. A so-called step-up approach has become an international standard, where at first transcutaneous and endoscopic catheter drainage are performed before increasing the operative trauma to video-assisted retroperitoneal necrosectomy or even open abdominal necrosectomy as a last measure. With this approach, morbidity and mortality could be drastically reduced^6^–^11^. Any intervention should only be performed after an initial stabilization period of about 3 weeks. The only exception should be limited to acute life-threatening conditions like bowel ischemia, abdominal compartment syndrome, or perforations^4^,^12^–^14^.

In 2012 our group reported a significant decrease in mortality if conservative treatment was consequently performed in patients with severe acute pancreatitis. For better understanding, we describe the patient’s cohorts, but for further details, we refer to our previous publication^4^. Before 2003 all patients in our department with severe necrotizing pancreatitis received fine-needle aspiration (FNA). The definitions of severe acute pancreatitis were based upon the original Atlanta classification^15^ since the revised version^1^ was published one year after our publication. When infection was proved, an aggressive open necrosectomy was performed. Twenty consecutive patients were analyzed as group 1. In 2003 treatment strategy was drastically changed, and all patients were treated conservatively for at least 3 weeks without performing FNA or any necrosectomy. The only exceptions were operations for acute emergencies like abdominal compartment syndrome, bowel ischemia, or bowel perforations. Twenty-four consecutive patients were analyzed as group 2. The severity of the disease did not differ between groups 1 and 2 with APACHE (Acute Physiology and Chronic Health Evaluation) II scores of 19.8 versus 16.1, Ranson core of 3.9 in both groups, and CTSI (Computed Tomography Severity Index) scores of 7.8 and 7.9, respectively. In group 2, all patients were treated according to the step-up approach, when indicated. The only indication for drain placement or further operations was the deterioration of patients despite ICU therapy. Only six patients received necrosectomy, treatment was performed mostly by interventional drainage only. These patients were analyzed as group 2a. Interestingly in group 2, nine patients did not receive any intervention at all – no drain placement nor any operation despite a severe course of the disease because they did not have drastic clinical deterioration. These nine patients were analyzed as group 2b. Between these two subgroups of patients, the severity of the disease was not different either. With the more conservative management, we reduced in-hospital mortality from 45% in group 1 to 8.3% in group 2.

In other words, in group 2, nine patients never had any interventions or operations, despite a prolonged need for intensive care unit treatment^4^. Therefore we do not have information about infection of pancreatic necrosis in these nine patients. In group 2a, three patients had sterile necrosis in all other patients, infection was proven. Since most patients were treated for more than 90 days, we only analyzed in-hospital mortality.

In the current cohort study, we performed a follow-up of the entire patient cohort to assess whether limited interventions could have negative effects in the years to follow.

## Materials and methods

We performed a follow-up of all patients treated in our department of surgery at a tertiary hospital for severe acute pancreatitis from 1996 to 2007. The short-term results of the change in treatment regimen were already published in 2012^4^. This work has been reported in line with the STROCSS (strengthening the reporting of cohort, cross-sectional and case–control studies in surgery) criteria^16^. This study is registered at Research Registry UIN researchregistry8697.

Follow-up was performed in different ways: Whenever possible, a telephone contact was established, followed by the mailing of a questionnaire and a consent form. If patients were already deceased, we gathered all information from the family practitioner and local registry offices. In these cases, informed consent was given by the family.

We especially collected information about the functional status of the pancreas – that is whether the patients indeed needed enzyme replacement therapy, need for peroral antidiabetic medication, or insulin replacement therapy. Prophylactic use of enzymes was categorized as having no need for therapy. Furthermore, we asked about habits like smoking or alcohol consumption and asked about the ability to work or whether acute pancreatitis had any influence on working status. Another focus was placed on recurrent hospitalization because of acute pancreatitis or whether further attacks needed medical treatment.

We were able to collect data from more than 90% of all patients and could therefore evaluate the outcome 10–22 years after initial treatment and discharge from our hospital with a median follow-up of 15 years.

Data analysis was ultimately performed with SPSS (Statistical Package for the Social Sciences) using *t* test; survival analysis was performed using Kaplan–Meier and log-rank tests were applicable. A *P* value of 0.05 or less was considered significant.

Considering the relatively low number of patients in the initial group, we could analyze long-term effects in 30 of 33 surviving patients. In every group, one patient refused to participate and one patient in group 2 was lost to follow-up.

In summary, 10 of 11 surviving patients of group 1 (90.9%) and 20 of 22 surviving patients of group 2 (90.9%) were included in this study. Eleven patients are included in group 2a and nine patients in group 2b. The initial group size (before treatment started) was 20 patients in group 1 and 24 patients in group 2. Details of patient population and interventions are published in the original study^4^.

## Results

### Recurrence and readmission due to acute pancreatitis

**Figure 1:**
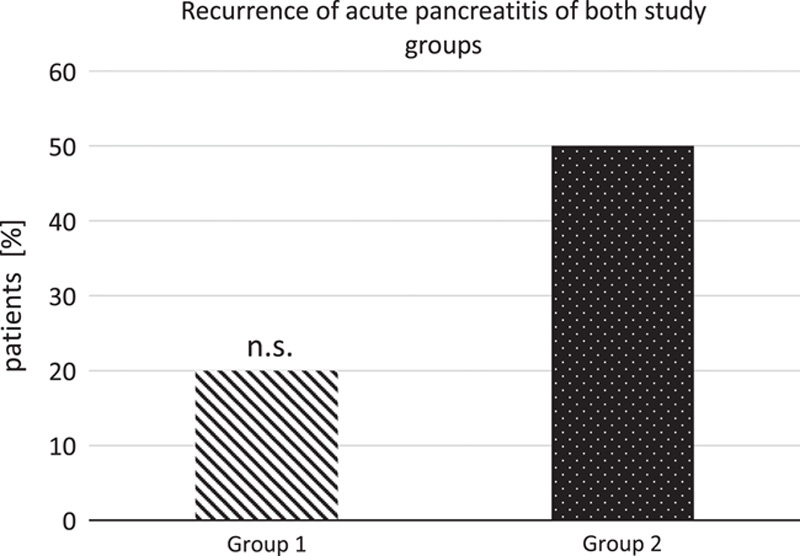
Recurrence and readmission of acute pancreatitis compared between study groups (group 1 early necrosectomy, *n*=10; group 2 primary conservative treatment, *n*=20), *P*=0.23. n.s., not significant.

In group 1, 2 out of 10 patients needed at least one readmission due to a recurrent attack of acute pancreatitis. In group 2, the proportion was higher with 10 out of 20. However, there was no statistically significant difference (*P*=0.23), see Figure 1.

**Figure 2:**
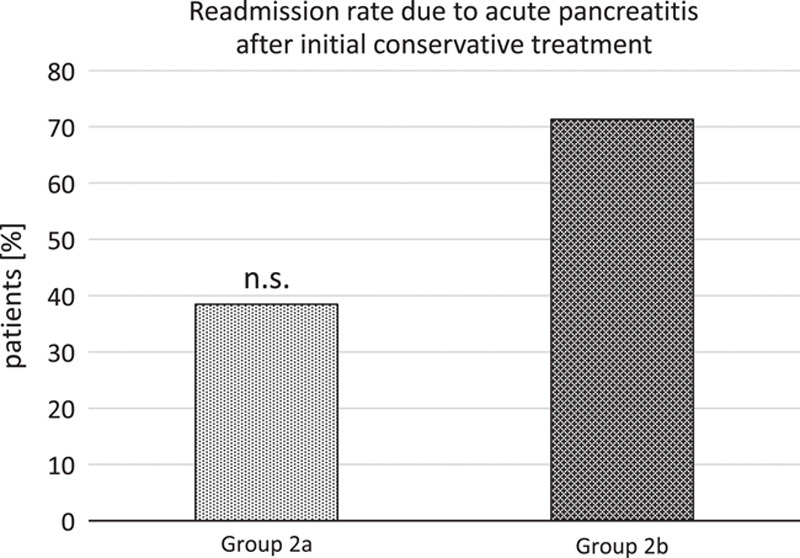
Recurrence and readmission due to acute pancreatitis after primary conservative treatment, no difference between subgroups (group 2a conservative treatment followed by step-up approach, *n*=11; group 2b conservative treatment and no intervention at all, *n*=9), *P*=0.3498. n.s., not significant.

Looking at the subgroups in group 2, those patients that never had any intervention (group 2b) had a higher proportion of readmissions than those treated by the step-up approach. However, there is again no statistically significant difference between groups (*P*=0.3498); see Figure 2.

### Long-term complications in conservatively treated patients (group 2)

Two patients in group 2 suffered from recurrent pain attacks in the years after the initial hospital treatment and attacks of pancreatitis due to a stenosis in the pancreatic duct and the common bile duct. Temporary stenting was necessary, but stents could be removed in the course of treatment. All other patients were treated symptomatically. No patient of group 2 needed surgical necrosectomy after initial discharge – not even those without any intervention at all.

### Endocrine insufficiency

**Figure 3:**
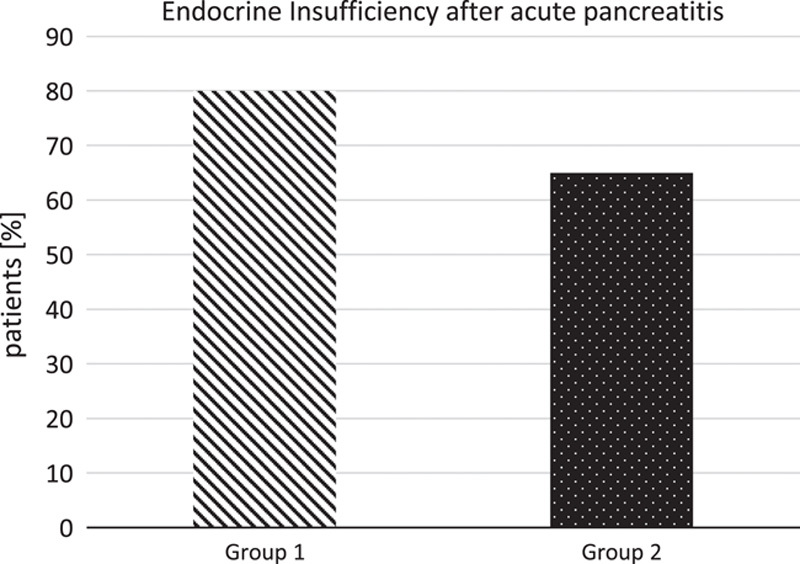
Rate of endocrine insufficiency (group 1 early necrosectomy, *n*=10; group 2 primary conservative treatment, *n*=20), *P*=0.78.

Throughout the entire cohort, there is a substantial proportion of patients with the need for antidiabetic medication. In group 1, there are 8 out of 10 with insulin-dependent diabetes mellitus. In group 2, there are 13 out of 20, who need medication; however, only two are sufficiently treated with oral antidiabetics alone, all others are insulin dependent. The rate of insulin-dependent diabetes in the surviving groups is 80% in group 1 and 55% in group 2. However, there is no statistical difference between the groups, see Figure 3.

Interestingly, subgroup analysis of group 2 revealed some differences: In subgroup 2a, there 6 out of 13 patients with diabetes, but in subgroup 2b, all patients developed diabetes, two were treated with oral antidiabetics, but the rest developed an insulin-dependent diabetes.

**Figure 4:**
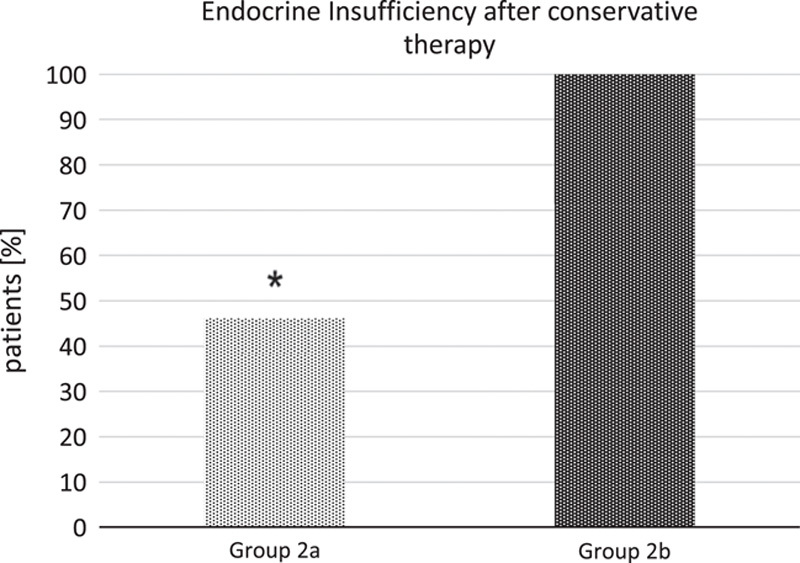
Rate of endocrine insufficiency after primary conservative treatment, group 2a conservative treatment followed by step-up approach, *n*=11 versus group 2b conservative treatment and no intervention at all, *n*=9, *P=0.044.

Comparing subgroups 2a versus 2b, there is a statistically significant difference with *P*=0.044. In other words, patients who did not receive any intervention at all during the initial treatment had a significantly higher rate of diabetes than those treated by the step-up approach, see Figure 4.

### Exocrine insufficiency

We defined exocrine insufficiency as the need for enzyme replacement therapy. There are no differences between the two study groups (*P*=1.0). In group 1, there are four patients, and in group 2, eight patients with the need for enzyme replacement. There are as well no differences in the subgroup analysis of group 2.

### Alcohol consumption

Alcohol consumption, in general, plays an important role in the pathogenesis of acute pancreatitis. After discharge, all patients were advised to refrain from alcohol consumption. In our follow-up, we found ongoing alcohol consumption in 30–45 % of the patients in group 1 versus 2 without any statistically significant difference.

### Ability to work

One important aspect of severe acute pancreatitis is the resulting reduced earning capacity or total disability. In our follow-up, there was no difference in the disease-related reduction in earning capacity. In group 1, six patients entered regular retirement, as did seven patients in group 2. Two and four patients are still working, and others suffer from disease-related reduction in earning capacity. Overall there was no difference between both groups.

### Long-term survival

**Figure 5:**
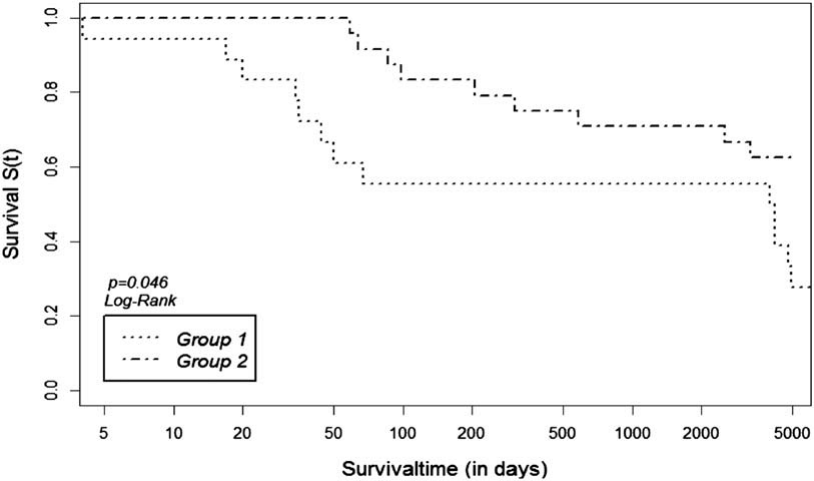
Long-term survival of both groups showing significantly better survival after conservative treatment (group 1 early necrosectomy, group 2 primary conservative treatment), *P*=0.049.

As published earlier^4^, there is a significant reduction in in-hospital mortality between groups with an impressive reduction of mortality in the conservatively treated group 2. In our follow-up, we can still demonstrate a survival advantage for these patients. In log-rank analysis, there is a statistically significant difference with *P*=0.049, see Figure 5. Interestingly, the subgroup analysis of group 2 revealed no difference either. Obviously, strict conservative management without any interventions does not result in any disadvantage for these patients.

## Discussion

Long-term data after conservative treatment of severe acute pancreatitis are rare. With this follow-up, we wanted to establish whether the burden of necrotic debris, which was not removed in many patients, could have a negative impact on long-term survival. Furthermore, untreated necrosis could lead to further complications or recurrent disease and hospitalization. Only a few studies have evaluated the long-term effects^17^–^26^. The main emphasis of these studies is different. Some studies looked at organ function, cost of treatment, and quality of life in mild and severe acute pancreatitis^17^–^19^,^26^. Others looked at sequelae of alcohol-induced pancreatitis^23^ or risk factors of mortality^24^. Only a few studies evaluated the long-term effects of conservative versus operative treatment. However, the follow-up periods vary from 18 to 86 months^18^,^20^ and are therefore shorter than the follow-up in our study, which in the median was 15 years.

Hollemans *et al*.^20^ could show that a step-up approach has long-term advantages compared with primary necrosectomy. The median follow-up time was 7 years. The step-up group of patients even had better endocrine and exocrine function. These results are similar to our results in a subgroup analysis of group 2. Patients who received interventions are comparable to the step-up group and also show better endocrine function. Chandrasekaran *et al*.^18^ had similar results but a shorter follow-up.

So far, there is no study we are aware of that has a longer follow-up than our current study (median follow-up 15±7 years), especially looking at conservative versus operative treatment and their long-term effects.

Certainly, the number of patients in our study is rather small. However, it is comparable to the above-mentioned studies, which included 35–73 patients^18^,^20^. Above all, we were limited to the original study population. Considering the small number of patients, it is important to mention that we at least were able to gather all relevant information of more than 90% of all patients in both groups.

Ahmed Ali *et al*. showed an increase in the risk of acute pancreatitis in patients who had previous attacks. Besides the recurrence of acute attacks, chronification also increases. However, this study does not compare different treatment strategies^27^. Additional risk factors for recurrence and chronification are alcohol and nicotine consumption^28^,^29^.

Our analysis did not show any disadvantages to conservative treatment with regard to the risk of recurrence. However, there is a trend towards an increase in recurrence in the subset of patients without any intervention. The difference is not statistically significant, which could be explained by the small sample size.

It is well known that the rate of recurrence increases when patients continue alcohol consumption^30^. In reverse, it has been shown that strict abstinence from alcohol can reduce recurrence as well as the rate of endocrine or exocrine insufficiency^31^,^32^.

In our study, we were not able to show any difference in the rate of alcohol consumption and, therefore, could not evaluate any effects of continued consumption. This is truly a weakness of our study design since we had to rely on voluntary information without any possibility of verification. This fact, combined with the low number of patients, unfortunately, leaves this question unanswered in our study.

In our follow-up, there was no difference in the disease-related reduction in earning capacity between the study groups. There are no data to compare our results. Most studies focus on the quality of life but do not compare different treatment strategies^17^,^21^,^33^.

Exocrine and endocrine insufficiency after severe acute pancreatitis has been reported in the literature with rates of diabetes of 40%^34^,^35^. Additionally, a few long-term follow-up studies report high risks of developing diabetes and exocrine insufficiency after acute pancreatitis^19^,^26^. However, long-term data about diabetes after primary conservative therapy is still very limited.

We could not see any difference in the rate of either endocrine or exocrine insufficiency between early necrosectomy or primary conservative treatment. However, in the group of primary conservatively treated patients, there was a higher incidence of diabetes when there was no intervention at all. Combining our results with other publications^18^,^20^, there is a clear advantage for primary conservative treatment of severe acute pancreatitis with a step-up approach where necessary. This can be performed endoscopically or surgically with equal results^10^,^36^. Furthermore, immediate drainage did not show superiority over postponed drainage^37^. However, based on our results, pure conservative treatment without any intervention at all is also a possible treatment option.

### Long-term survival

There are not much data available about long-term survival comparing different treatment strategies in severe acute pancreatitis.

There are a few studies evaluating long-term mortality, but these focus either on risk factors^38^–^40^ or the influence of special interventional or operative methods^41^,^42^. Husu *et al*.^38^ performed a retrospective study and evaluated short-term and long-term survival. They could show a survival benefit for patients younger than 60 years but did not differentiate between different treatment strategies. Negative risk factors for survival after acute pancreatitis are alcohol, older age, as well as endocrine insufficiency^24^,^25^. Additionally, comorbidities, as well as multiple organ failure during the course of acute pancreatitis, are associated with reduced long-term survival^25^,^40^.

In the current study, we could demonstrate a significant difference in long-term survival in favor of primary conservative treatment of severe acute pancreatitis. So far, a short-term benefit has been known, though we are now able to show a long-term benefit for the first time.

## Conclusion

So far, there is scarce data about long-term survival after conservative treatment of severe acute pancreatitis. Therefore even data from studies with small numbers of patients are of great importance.

Our study demonstrates positive long-term effects, even after a median follow-up of 15 years, if patients are treated primarily conservatively. Therefore, the combination of initial strict conservative management followed by the classical step-up approach seems to be the most effective treatment regimen for this disease.

Primary conservative treatment of severe acute pancreatitis is a safe option and poses no risk of clinical problems. Based upon our data, there is no absolute need for necrosectomy in severe acute pancreatitis.

## Ethical approval

Approved by the ethics committee of the University of Rostock A 2015-0064.

## Consent

Informed consent was obtained from all participants in the study.

## Sources of funding

No funding was received for this study.

## Author contribution

G.A.: study design, data acquisition, analysis of data, and writing of the manuscript; E.K.: study design, analysis of data, and proofreading of the manuscript; J.F.: data acquisition and analysis of data; C.S.: interpretation of data and proofreading of the manuscript.

## Conflicts of interest disclosure

There are no conflicts of interest.

## Research registration unique identifying number (UIN)

1. Name of the registry: Research Registry.
2. Unique identifying number or registration ID: researchregistry8697.
3. Hyperlink to your specific registration (must be publicly accessible and will be checked): not applicable.

## Guarantor

Guido Alsfasser.

## Provenance and peer review

Not commissioned, externally peer-reviewed.
